# Historical Case of Cervical Penetrating Wound: From First Aid to Surgical Intervention

**DOI:** 10.1155/2017/2415679

**Published:** 2017-10-22

**Authors:** Koné Fatogoma Issa, Keïta Mohamed Amadou, Soumahoro Siaka, Konaté N'faly, Diarra Kassim, Timbo Samba Karim

**Affiliations:** Department of ENT Head and Neck Surgery, Teaching Hospital Gabriel Toure, Bamako, Mali

## Abstract

**Objective:**

We report a case of cervical penetrating wound by posing the problem of its support and by analyzing the chain of survival of a patient to human sacrifice.

**Case Report:**

It was an 11-year-old boy admitted to the hosting service of cervical penetrating wound emergency occurring in a context of human sacrifice by weapon (knife). On admission, the conscious patient had a left cervical hematoma at the level of the cervical zone II and severe signs of acute anemia. The exploratory cervicotomy, carried out 12 hours after the trauma under transfusion, allowed us to highlight a section of the front edge of the sternocleidomastoid and previous jugular muscles under hyoid. We noted the presence of a linear wound of 1 cm at the level of the left internal jugular vein. The wound of the internal jugular vein has been repaired with the Prolene 4.O. The outcome was good, allowing the exit 10 days after cervicotomy.

**Conclusion:**

The causal circumstances of cervical penetrating wounds are diverse. Their importance or their severity depends on the causative circumstances dominated by aggression and attempts to autolysis. Human sacrifice, with use of the weapon, is an exceptional circumstance.

## 1. Introduction

The nonballistic penetrating neck injuries are potentially life-threatening emergencies with an incidence of 1-2/100000 among all neck traumas [1]. These particular traumatic neck injuries constitute, in our context of exercise, a problem of the efficient integration of ENT emergencies into the overall system of emergencies [2]. The severity of these wounds depends on the etiological circumstances [3]. They varied and are dominated by assaults and attempts at autolysis, with the weapon as the main vulnerable agent [4, 5]. The human sacrifice with use of the weapon and survival of the patient constituted a particular circumstance of cervical penetrating wound in this observation that we report. In this case, we also address the issue of the chain of care for this serious ENT emergency.

## 2. Case Report

**Figure 1:**
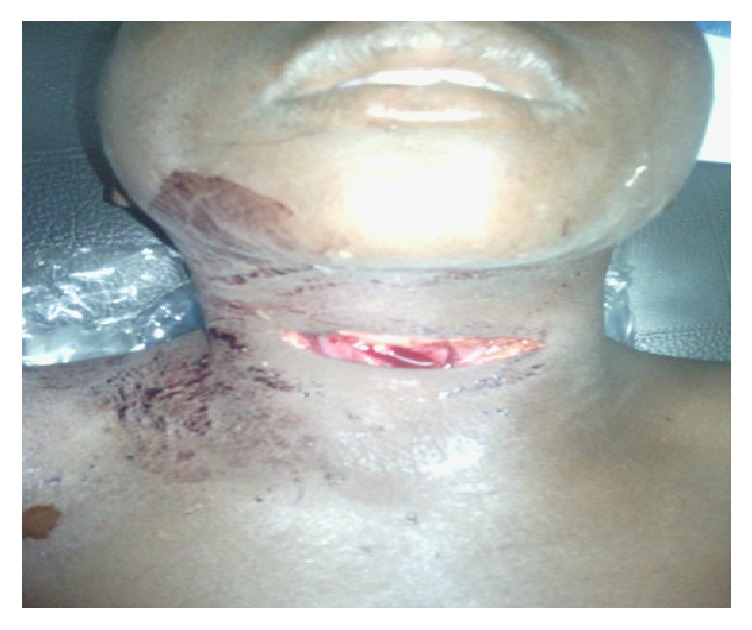
Horizontal basal-cervical penetrating wound.

**Figure 2:**
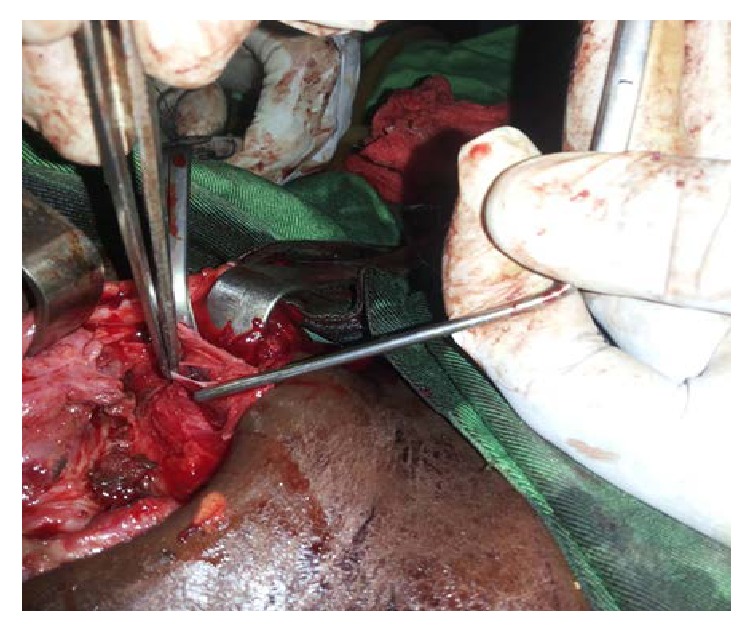
Wound of the left internal jugular vein between two vascular clamps, edges separated by dissecting forceps without claw.

**Figure 3:**
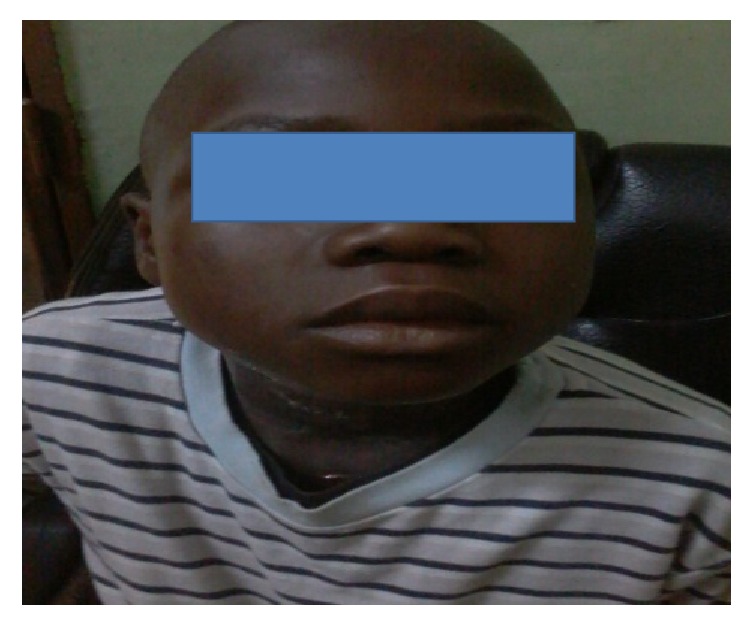
Patient after operation.

The patient, named MC, aged 11, was admitted to the emergency department for a left anterior cervical penetrating wound. He was allegedly the victim of a stabbing attempt during a human sacrifice. Having escaped from his tormentor, he was sent to the emergency department by a rescue team warned by a motorcyclist who had seen him on the roadside. Upon arrival at the emergency department, the child had a hemorrhagic shock with hemoglobin and hematocrit estimated at 5 g/dl and 15%, respectively. Physical examination of the wound revealed a horizontal linear penetrating wound (Figure 1), which was about 6 cm long and was located in zone II with a large lateral left hematoma. The decision of exploratory and repair cervicotomy was made after transfusion of two iso group iso rhesus blood units and 12 hours after the trauma. At exploration, we noted the presence of a left cervical hematoma. Compression with the finger facilitated the clamping of the bleeding zone and allowed a dissection from bottom to top of the left internal jugular vein. This procedure allowed the detection of wound on the inner jugular vein measuring 1 cm in length (Figure 2). The haemostasis was perfect after suturing in single stitches separated with the Prolene 4.0. The closure was done under a drain, after repair of the muscles. Antibiotics based on amoxicillin-clavulanic acid were introduced for 10 days, parenterally. The operative sequences were simple (Figure 3); the outcome was good, allowing the exit 10 days after cervicotomy.

## 3. Discussion

In a context of undermedicalization, the integration of major ENT emergencies into the general emergency system encounters many difficulties [2]. The cervical penetrating wounds, to which our case is related, are an illustration. Problems are encountered at two levels, before admission to hospital or prehospital care and once the patient is admitted to the hospital. Before admission to the hospital, it is the first useful and necessary first aid of resuscitation and practical gesture like compression, in front of any externalized brutal hemorrhage. Prehospital care can be provided by a team of rescuers who are aware of the rules in this area or by a medical team that is trained in this task. Jakubowski et al. [6–8], in these studies, enact the chain of care. Although we saved the child, adequate prehospital management was lacking in our patient, admitted to the hospital in a nonmedical vehicle. In the penetrating wounds of the neck, the most vulnerable agent is usually represented by the white weapon [4]. In our case, the particularity was the context of human sacrifice, with use of the knife. The possibility of very serious, vascular, respiratory, digestive, and neurological injuries that could immediately put life-threatening factors at risk was the big obsession here. The delay of exploratory cervicotomy was 35 hours in the series of Tall et al. [2] and 12 hours in this observation. This long period of cervicotomy could have been fatal to the patient. Exploratory cervicotomy was indicated in front of an open wound and a hemodynamically unstable patient. This therapeutic principle corroborates those of several authors [1, 5]. The wound in the jugular vein was successfully repaired. In front of a penetrating wound of the neck, the following diagram is enacted: call of the center of assumption, dispatch of a medical team, and preparation of the hospital hospitality. The information of head and neck surgeons and, if necessary, of vascular surgeons will be necessary in the presence of signs pointing towards a vascular wound. In the case of our patient, although the call of the emergency team by the motorcyclist played a preponderant role, as well as the sending of a rescue vehicle, he missed medical procedures such as compression and resuscitation. The providential haemostasis performed by the hematoma, the resuscitation at the admission, and the well-conducted cervicotomy allowed in this observation saving the small patient.

## 4. Conclusion

The management of penetrating neck wounds is an illustration of the difficulties of efficient integration of major ENT emergencies into the general emergency system. The patient's journey, from the first measures of first aid to explorative cervicotomy, must be the subject of a protocol codifying the management. This would reduce morbidity and mortality.
